# The effects of exposure to pyriproxyfen and predation on Zika virus infection and transmission in *Aedes aegypti*

**DOI:** 10.1371/journal.pntd.0008846

**Published:** 2020-11-17

**Authors:** Abdullah A. Alomar, Bradley H. Eastmond, Barry W. Alto

**Affiliations:** University of Florida, Department of Entomology and Nematology, Florida Medical Entomology Laboratory, Vero Beach, Florida, United States of America; University of Glasgow, UNITED KINGDOM

## Abstract

Zika virus (ZIKV) is an emerging mosquito-borne pathogen that can cause global public health threats. In the absence of effective antiviral medications, prevention measures rely largely on reducing the number of adult mosquito vectors by targeting juvenile stages. Despite the importance of juvenile mosquito control measures in reducing adult population size, a full understanding of the effects of these measures in determining mosquito phenotypic traits and in mosquito-arbovirus interactions is poorly understood. Pyriproxyfen is a juvenile hormone analog that primarily blocks adult emergence, but does not cause mortality in larvae. This mechanism has the potential to work in combination with other juvenile sources of mortality in nature such as predation to affect mosquito populations. Here, we experimentally evaluated the effects of juvenile exposure to pyriproxyfen and predatory mosquito *Toxorhynchites rutilus* on *Aedes aegypti* phenotypes including susceptibility to ZIKV infection and transmission. We discovered that combined effects of pyriproxyfen and *Tx*. *rutilus* led to higher inhibition of adult emergence in *Ae*. *aegypti* than observed in pyriproxyfen or *Tx*. *rutilus* treatments alone. Adult body size was larger in treatments containing *Tx*. *rutilus* and in treatments mimicking the daily mortality of predation compared to control or pyriproxyfen treatments. Susceptibility to infection with ZIKV in *Ae*. *aegypti* was reduced in predator treatment relative to those exposed to pyriproxyfen. Disseminated infection, transmission, and titers of ZIKV in *Ae*. *aegypti* were similar in all treatments relative to controls. Our data suggest that the combination of pyriproxyfen and *Tx*. *rutilus* can inhibit adult *Ae*. *aegypti* emergence but may confer a fitness advantage in survivors and does not inhibit their vector competence for ZIKV relative to controls. Understanding the ultimate consequences of juvenile mosquito control measures on subsequent adults’ ability to transmit pathogens is critical to fully understand their overall impacts.

## Author summary

Mosquito control approaches primarily depend on lowering the number of potential adult mosquito vectors by inhibiting juvenile stages to reduce the risk of pathogen transmission. Pyriproxyfen is a juvenile hormone analog that inhibits the emergence of adult mosquitoes by interrupting metamorphosis, but does not target larvae. This mechanism allows natural sources of mortality like predation to act in combination with pyriproxyfen to affect mosquito population size. Here, we determined the effects of juvenile exposure to pyriproxyfen and predatory mosquito *Toxorhynchites rutilus* on adult *Aedes aegypti* traits, including infection with Zika virus. Combined effects of pyriproxyfen and *Tx*. *rutilus* led to strong inhibition of adult emergence in *Ae*. *aegypti*. Treatments containing predators or those mimicking the daily mortality of predation produced larger sized adults. Susceptibility to ZIKV infection was lowest in the predator treatment and highest in the pyriproxyfen treatment. Disseminated infection, transmission, and viral titers of ZIKV were similar between treatments. Our data suggest that the combination of pyriproxyfen and predators can enhance inhibition of adult *Ae*. *aegypti* emergence, but survivors may have fitness benefits such being larger mosquitoes. Understanding the consequences of control approaches in mosquito-pathogen interactions will assist to evaluate their suitability in mosquito control programs.

## Introduction

Zika virus (ZIKV) is an emerging infectious pathogen that causes public health issues in many regions of the world. Zika virus (family: *Flaviviridae*, genus: *Flavivirus*) is an enveloped, positive-sense, single-stranded RNA virus with approximately 11,000 nucleotides. First isolation of ZIKV was in 1947 from serum of a non-human primate rhesus monkey *Macaca mulatta* stationed in the Uganda's Zika Forest [1,2], and later ZIKV was isolated from a wild-caught mosquito *Aedes africanus* in 1948. Early cases of ZIKV in humans were reported in 1952 by serological surveys in eastern Nigeria and Uganda [3,4]. Although primarily a mosquito-borne agent, ZIKV may be transmitted sexually [5], through blood transfusion [6], and from mother-to-child [7,8], modalities which further complicate control strategies and ZIKV epidemiology [2,9]. Viral infection in human usually results in mild symptoms; however, ZIKV has been implicated in neurological complications resulting in Guillain-Barré syndrome (i.e., acute inflammatory polyneuropathy) and microcephaly (i.e., severe decrease in the head circumference) in newborn babies, making it a serious public health threat [10–13].

*Aedes aegypti* is the primary vector of ZIKV and several neglected arthropod-borne viruses (arboviruses) that affect human health, including dengue virus (DENV) and chikungunya virus (CHIKV) [14]. Originating from Africa, *Ae*. *aegypti* is now widely spread in tropical, subtropical, and some temperate areas around the world [15,16]. The invasive success of *Ae*. *aegypti* is largely attributable to its life-history characteristics which exploit human-dominated habitats. *Aedes aegypti* has adapted to use domestic and urban environments, where larvae dwell in man-made containers such as vases, plastic containers, tires, gutter eaves, tubs, and cisterns [17]. Females of *Ae*. *aegypti* show a preference for blood feeding on humans, a behavior which contributes to its primary role as an arbovirus vector, and gonotrophic discordance where females may ingest more than one blood meal per gonotrophic cycle, allowing for increased probability of acquiring and transmitting pathogens and associated disease incidence [18,19,20,21]. Additionally, this species exhibits skip oviposition, whereby females oviposit eggs in more than one container during a gonotrophic cycle, a hedge betting strategy [22]. Eggs of the container-dwelling *Ae*. *aegypti* are more tolerant to desiccation than other mosquitoes (e.g., *Anopheles* and *Culex*), a trait which enables eggs to resist unfavorable environmental conditions without losing their viability [23].

Since there are no effective antiviral drugs or vaccines for most arboviruses, including ZIKV, control of vectors is the primary method used to combat ZIKV transmission. Mosquito control programs have extensively relied on the use of insecticides (e.g., larvicides) against juvenile stages to suppress potential adult vectors [24,25]. Insect growth regulators (IGRs) are chemical substances that disrupt the development and growth of mosquitoes and provide an alternative to larvicides for controlling *Ae*. *aegypti*, especially among geographic populations that exhibit resistance to insecticides, particularly organophosphates and pyrethroids [24–27]. Pyriproxyfen is a synthetic analog of juvenile hormone, a natural IGR in insects, that disrupts growth and development during the juvenile stages. Pyriproxyfen primarily interferes with metamorphosis at the end of pupal development [24,28]. Pyriproxyfen and other IGRs inhibit metamorphosis to adult stages after a short time exposure at exceptionally low concentrations making them favorable options for mosquito control [29–31]. Additionally, pyriproxyfen is effective against many insects of public health importance and is recommended for controlling mosquitoes by the World Health Organization Pesticide Evaluation Scheme [32–34].

Novel strategies in how pyriproxyfen is utilized has further enhanced its potential as a tool for mosquito control. Auto-dissemination of pyriproxyfen is a promising technique for mosquito control, where pyriproxyfen can be mechanically disseminated to new oviposition sites to interrupt metamorphosis of juvenile mosquitoes by females that previously visited pyriproxyfen-treated stations or mated with contaminated males [35,36]. Pyriproxyfen may further mitigate risk of pathogen transmission through morphological and physiological aberrations among adult and juvenile mosquitoes that come in contact with pyriproxyfen [37]. For example, short exposure to pyriproxyfen induced reproductive disruption and lifespan reduction in adult mosquitoes surviving the exposure [38,39]. Also, juvenile exposure to pyriproxyfen caused damage in midgut cells of *Ae*. *aegypti* larvae compared to unexposed controls [40]. These observations suggest that exposure to pyriproxyfen during the juvenile stages may have transstadial effects and alter fitness-related traits, mosquito immunity, susceptibility to infection, and transmission of pathogens among adult survivors, as observed with other insecticides [41,42].

The mode of action of pyriproxyfen which targets pupal-adult transformation takes advantage of other natural sources of mortality among mosquito larvae, especially density-dependent mortality, a strong regulator among container inhabiting mosquitoes [43]. For instance, predation, nutrient deprivation, and intra- and interspecific competition from larval crowding may cause mortality in mosquito larvae before pyriproxyfen-induced pupal mortality and so may allow for greater inhibition of adult emergence. This assumption predicts that pyriproxyfen and other natural sources of mortality (e.g., biological control agents such as predators) act in conjunction to inhibit pathogen transmission by reducing the number of adult mosquitoes.

Biological control approaches that exploit predatory species have historically been applied to reduce pathogen transmission risk by inducing juvenile mortality and inhibiting recruitment to the adult stage [44]. The predatory mosquito larvae of *Toxorhynchites rutilus* have been used in biological control trials against several mosquitoes, including *Ae*. *aegypti*, with mixed success due to their voracious appetite and shared habitat with other container mosquitoes [45,46]. The use of *Tx*. *rutilus*, and other biological control agents, in combination with pyriproxyfen may exacerbate mortality in *Ae*. *aegypti* since the latter has the desirable feature of not targeting the predaceous stage (i.e., larvae) of *Tx*. *rutilus*. In addition to the direct effects of prey consumption by predators, several studies indicated that prey exposure to stress of predators can induce sublethal costs in their prey in the form of indirect effects on behavior, physiology, and morphology [47–51]. For example, exposure to predatory dragonfly nymph during the juvenile stages modified adult *An*. *gambiae* susceptibility to fungus parasites [52]. The exposure of juvenile *An*. *coluzzii* to a predatory backswimmer led to alterations in adult traits, including size and fecundity [53]. Also, exposure to predatory *Tx*. *rutilus* during juvenile stages decreased lifespan of adult survivors in *Ae*. *aegypti*, suggesting an additional benefit to mitigating arbovirus transmission risk [54]. These results suggest that the exposure to predators such as *Tx*. *rutilus* may indirectly influence phenotypic traits, including immunity, of surviving adult mosquitoes and therefore alter their susceptibility to pathogen infection.

Taken together these findings suggest that exposure to pyriproxyfen and *Tx*. *rutilus* may alter arbovirus transmission directly by reducing the number of emerged adults and indirectly by modifying traits of surviving adults and their susceptibility to pathogen infection. The purpose of our study was to assess the effects of exposure to pyriproxyfen independently or in combination with mosquito predator *Tx*. *rutilus* on inhibition of adult emergence, body size, and mosquito-arbovirus interactions. Specifically, we measured susceptibility to ZIKV infection, disseminated infection, transmission, and viral titers in *Ae*. *aegypti* following orally ingestion of ZIKV.

## Methods

### Ethics statement

Zika virus (Asian lineage, strain PRVABC59, GenBank number KU501215) used in this research was originally isolated from serum of an infected human patient in Puerto Rico in 2015. An isolate of ZIKV was provided by the U.S. Centers for Disease Control and Prevention (Division of Vector-Borne Diseases, Arboviral Diseases Branch). Zika virus propagation, infectious blood meal preparations, and experimental infections of adult mosquitoes were performed in an arbovirology research facility at the Florida Medical Entomology Laboratory (BSL2+ and ACL2+) in accordance with the approved protocol by the University of Florida’s Institutional Biosafety Committee and Institutional Animal Care and Use Committee.

### Mosquitoes

*Aedes aegypti* (F2 generation) used in this research was from field collections made in Vero Beach, FL. Larvae (F2) were provided with food consisting of equal parts lactalbumin and *Saccharomyces cerevisiae* yeast. Newly pupated mosquitoes were transferred to plastic cups filled with water and held in adult rearing cages (30 x 30x 30 cm, BioQuip Products, Rancho Dominquez, CA) for adult emergence. Adult mosquitoes were provided with constant access to 10% sucrose solution *ad libitum* through cotton wicks. Mosquitoes were reared in a bioroom at controlled conditions: 60–80% relative humidity, 28°C±1°C, and 14:10-h light: dark photoperiod diurnal cycle.

Eggs of laboratory strain of predatory mosquito *Tx*. *rutilus* were obtained from Lee County Mosquito Control District in Lehigh Acres, FL. The colony of *Tx*. *rutilus* was held in a cage (65 x 37 x 50 cm) and maintained in an insectary core facility with natural light and photoperiod. Adults had constant access to 10% sucrose solution from moistened cotton and oviposition cups filled with water. Since females of *Tx*. *rutilus* lay fertile eggs autogenously, newly hatched larvae were collected from oviposition cups and transferred to small cell trays to prevent cannibalism (1 larva per cell). Larvae of *Tx*. *rutilus* were fed *Ae*. *aegypti* larvae every two days until pupation after which they were transferred into the colony cage for adult emergence.

### Pyriproxyfen preparation

**Fig 1:**
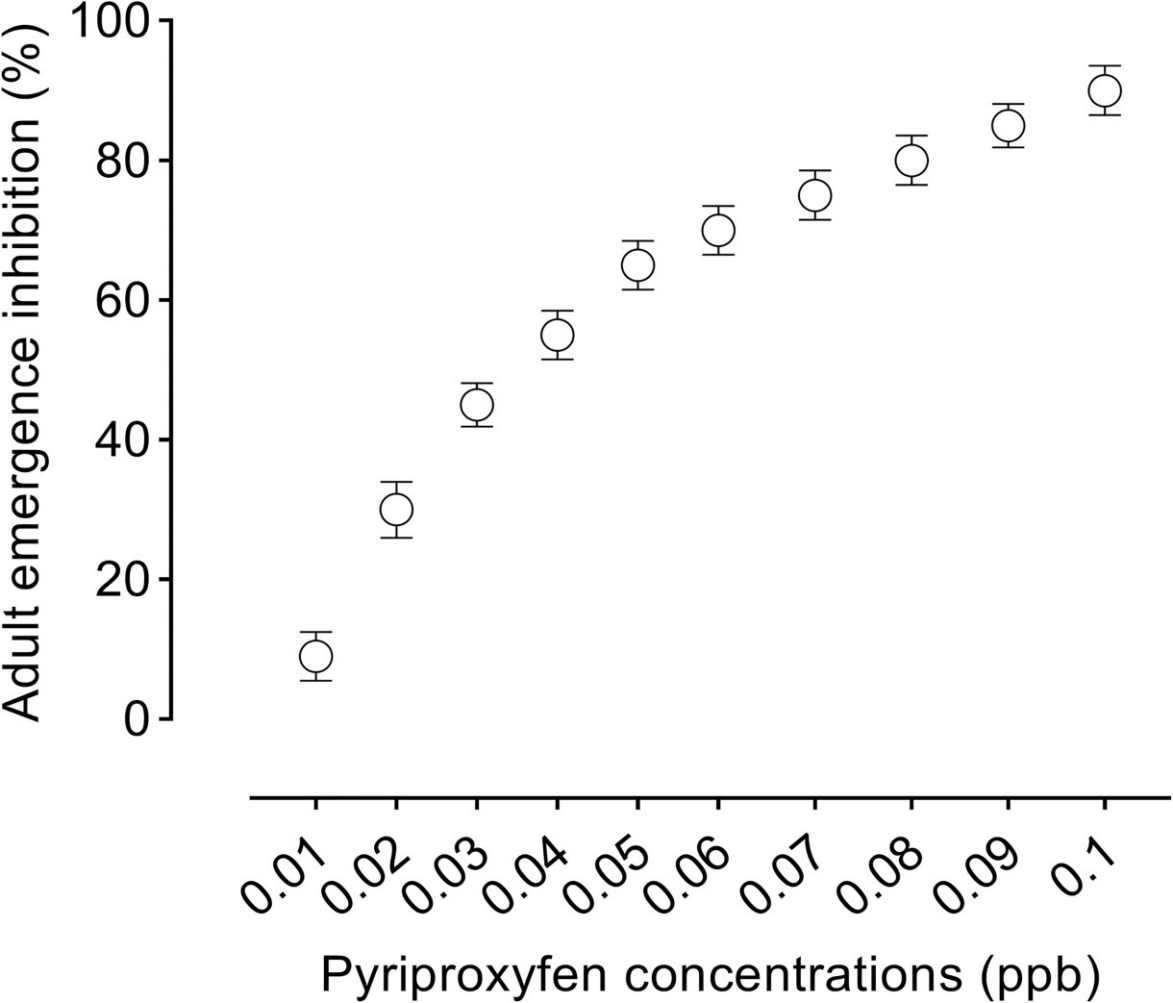
Adult emergence inhibition in *Ae*. *aegypti* in response to pyriproxyfen concentrations.

A stock solution of juvenile hormone analog pyriproxyfen (10 ppb) (2-[1-Methyl-2-(4-phenoxyphenoxy) ethoxy] pyridine—Nyguard IGR) was prepared in tap water and serial diluted to assess its toxicity to *Ae*. *aegypti*. Based on our preliminary toxicity assessment, a single low concentration of pyriproxyfen (0.022 ppb) that causes 30% inhibition of adult emergence was used in our experiments (Fig 1). Exposure to low concentration of pyriproxyfen was expected to induce direct and indirect effects on *Ae*. *aegypti* and still allow for enough survivors to assess their susceptibility to ZIKV infection, especially in treatments where mortality is anticipated to be higher than other treatments (e.g., pyriproxyfen+predator).

### Juvenile treatment manipulation

**Fig 2:**
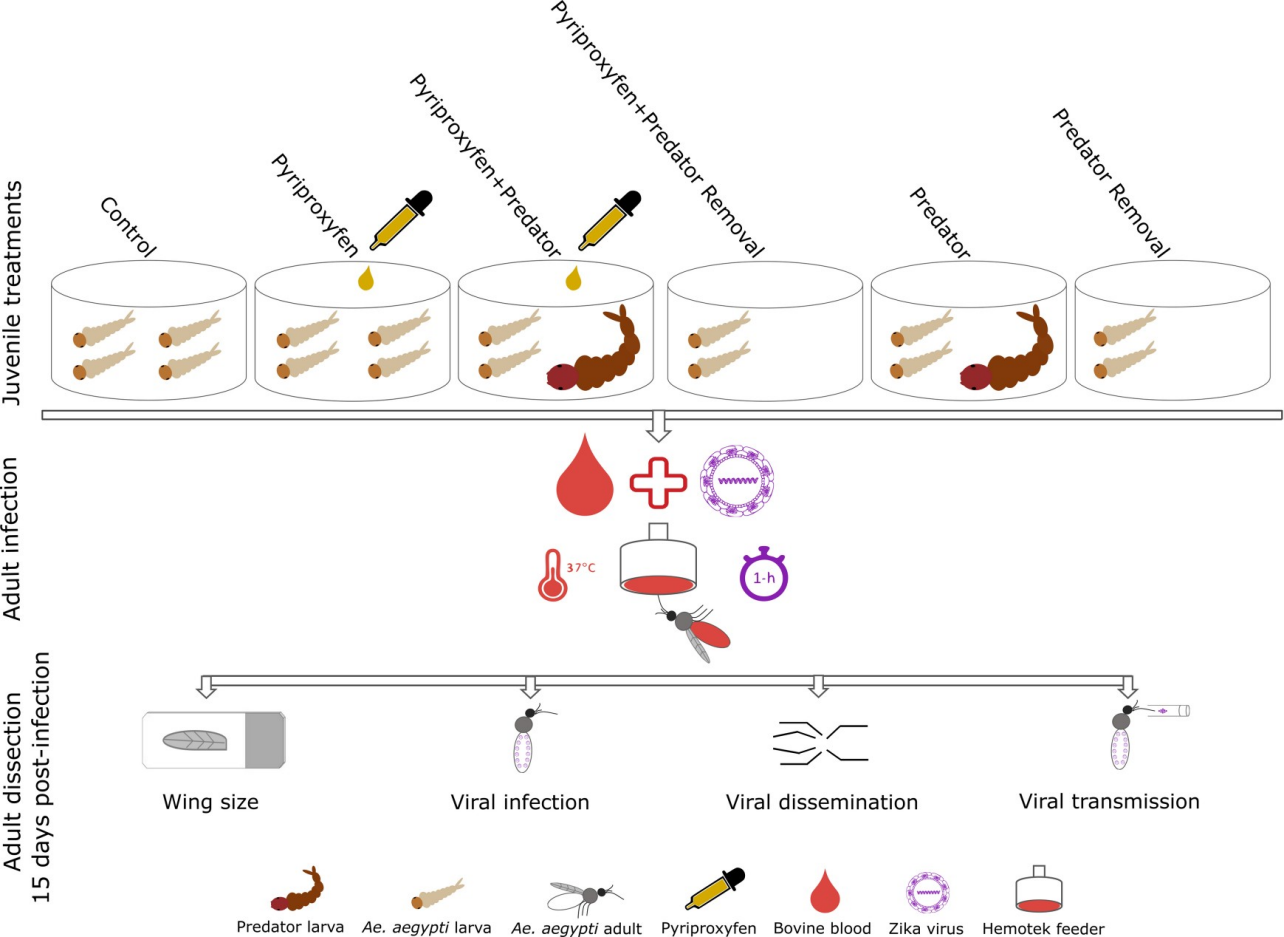
Schematic diagram illustrating the experimental design.

Three hundred newly hatched first-instar larvae of *Ae*. *aegypti* were placed into each experimental pan (experimental unit) containing 1.5 L of water and 0.2 g of larval food. Experimental pans were assigned to one of the following treatment groups: control, pyriproxyfen, pyriproxyfen+predator, pyriproxyfen+predator removal, predator, predator removal (Fig 2). Each of the six treatments was replicated five times for a total of 30 experimental units. After the addition of *Ae*. *aegypti* larvae to the experimental pans, a first-instar larva of *Tx*. *rutilus* was introduced to each of the replicates of pyriproxyfen+predator and predator treatments. Once *Ae*. *aegypti* developed to third-instar, a low concentration of pyriproxyfen (0.022 ppb) was applied to each of the replicates of pyriproxyfen+predator and pyriproxyfen treatments. For treatments that contain a predator (e.g., pyriproxyfen+predator and predator), total number of *Ae*. *aegypti* prey was counted daily using established methods [54,55]. On a daily basis, the number of consumed or dead *Ae*. *aegypti* prey in the pyriproxyfen+predator and predator treatments was averaged across all replicates as a measure of mortality rate and then removed from pyriproxyfen+predator removal and predator removal treatments, respectively as described elsewhere [54]. No treatment manipulations were made for the controls in which larvae were not exposed to pyriproxyfen or *Tx*. *rutilus*. Pupae of *Ae*. *aegypti* from experimental pans were collected daily and transferred to plastic cups and placed in cages, by treatment group and replicate, to capture newly emerged adults. Emergence of adults was measured for each experimental pan as the total number of adults eclosed divided by the number of larvae originally added to each pan, expressed as a percent. All experimental pans were held under controlled environmental conditions in bioroom maintained at a 14:10 light: dark photo regime, 28°C±1°C and 60–80% humidity.

### Zika virus propagation

Mammalian host cells and ZIKV were cultured and maintained in growth media (HyClone, Medium 199, GE Healthcare, Logan, UT), supplemented with 10% fetal bovine serum, antibiotics (penicillin/streptomycin), and mycostatin. Confluent monolayers of African green monkey epithelial (Vero) cells (175 cm^2^) were inoculated with 500 μL of diluted ZIKV stock at multiplicity of infection of 0.1 and incubated at 37°C and 5% CO_2_ atmosphere for 1-h to initiate viral infection, after which 24-mL of media (M199) were added and incubated further until cytopathic effects observed after six days [56]. Freshly harvested media from ZIKV-infected cell cultures were combined with defibrinated bovine blood (Hemostat Laboratories, Dixon, CA) and adenosine triphosphate (0.005 M) before the start of feeding trials for the ZIKV infection study.

### Zika virus infection

Seven-to ten-day-old adult females were transferred to paperboard cages (10 x 10 x 7 cm) and starved from sucrose solution but not water for 24-h prior to viral infection feeding trials. Mosquitoes were allowed to feed on ZIKV-infected blood meals using Hemotek membrane feeders (Discovery Workshops, Lancashire, UK) warmed to 37°C for 1-h. Following the feeding trials, mosquitoes were anesthetized with CO_2_ for sorting and fully engorged blood-fed females were returned to cages with continuous access to 10% sucrose solution via cotton pads and held at the same conditions as rearing the juvenile stages for a 15-day ZIKV incubation period. Aliquots of 1 mL of ZIKV-infected blood were taken after blood feeding trials and placed into 2 mL of cryogenic vials (MilliporeSigma, Burlington, MA) and stored at -80°C for determination of viral titers. The ZIKV titers in blood meals were 6.7±0.3 log_10_ plaque-forming unit equivalents per mL.

### Zika virus transmission

Fifteen days post-infection, mosquitoes were anesthetized with CO_2_ for dissection. Legs with a single wing were removed from the rest of the body. Saliva was collected by forced salivation by inserting the proboscis of each mosquito into a microhematocrit capillary tube (Thermo Fisher Scientific, Waltham, MA) containing type B immersion oil (Cargille Laboratories, Cedar Grove, NJ). Mosquitoes were allowed to salivate for approximately 1-h, after which the saliva and oil were expelled under pressure into microcentrifuge tubes containing 300 μL of incomplete media (M199). Tests of mosquito saliva are a proxy for the ability of a mosquito to transmit virus by bite. Bodies and legs were separately placed into microcentrifuge tubes (Thermo Fisher Scientific, Waltham, MA) containing 1 mL of incomplete media (M199). All bodies, legs, and saliva samples from mosquitoes were frozen at -80°C until further processing.

### Viral nucleic acid extraction and quantitative RT-PCR

Body and legs of mosquitoes were thawed then homogenized using a TissueLyser II automation system (Qiagen, Hilden, Germany) at 19.5 Hz for 3 min and centrifuged for 5 min at 13,200 rpm. Viral nucleic acids were extracted from mosquito body, legs, and saliva using the QIAamp Viral RNA Mini Kit (Qiagen, Valencia, CA) and eluted in 60μL of buffer according to the manufacturer's instructions. Zika virus RNA in mosquito samples was determined using the Superscript III One-Step Quantitative RT-PCR System with Platinum Taq kit (Invitrogen, Carlsbad, CA) with the C1000 Touch Thermal Cycler, CFX96 Real-Time System (Bio-Rad Laboratories, Hercules, CA). Zika virus primers and probes used in this experiment were synthesized by Integrated DNA Technologies (Coralville, IA) with the following sequences (forward primer: 5′-CTTCTTATCCACAGCCGTCTC-3′; reverse primer: 5′-CCAGGCTTCAACGTCGTTAT-3′; and probe: 5′-/56-FAM/AGAAGGAGACGAGATGCGGTACAGG/3BHQ_1/-3′). The program for quantitative RT-PCR consisted of 2 min at 94°C, 12 sec at 94°C, 30 min at 50°C, 1 min at 58°C linked to 39-cycles. Water and ZIKV RNA stock were used in each reaction run as a negative and positive control standard, respectively. To quantify viral titration of ZIKV in mosquito body, legs, and saliva, a standard curve was prepared to relate the amount of ZIKV RNA detected in mosquito samples to serial dilutions of ZIKV stock with plaque assays, expressed as plaque forming unit equivalents per mL (PFUE/mL) [57]. Infection rate was determined by the percent of females with ZIKV RNA-positive bodies from the total number that fed on the infectious blood meal. Disseminated infection and transmission rates were determined by the percent of females with infected bodies that have ZIKV RNA-positive legs and saliva, respectively [58].

### Body size determination

Juvenile treatment effects on female size were determined by measuring wing length as a proxy for size of the body [59,60]. A single wing was dissected from each ZIKV-infected female and mounted on double sided tape on glass microscope slides (Cardinal Health, Dublin, OH). Wing length was measured in millimeters from axillary margin to the apical notch without considering wing fringe using computer imaging software (IMT i-Solution lit, Princeton, NJ).

### Statistical analysis

Juvenile treatment manipulation effects on *Ae*. *aegypti* traits (adult emergence and female wing length) and ZIKV infection measurements (infection, disseminated infection, transmission, and viral titers) were analyzed using separate analysis of variance tests (ANOVAs) and Tukey’s multiple comparisons adjustment. Canonical-correlation analysis was performed to determine the overall relationship between *Ae*. *aegypti* traits and vector competence measurements for ZIKV [61]. A *p*< 0.05 was considered statistically significant. All statistical analyses were performed using SAS software [62].

## Results

### Adult emergence and body size

**Fig 3:**
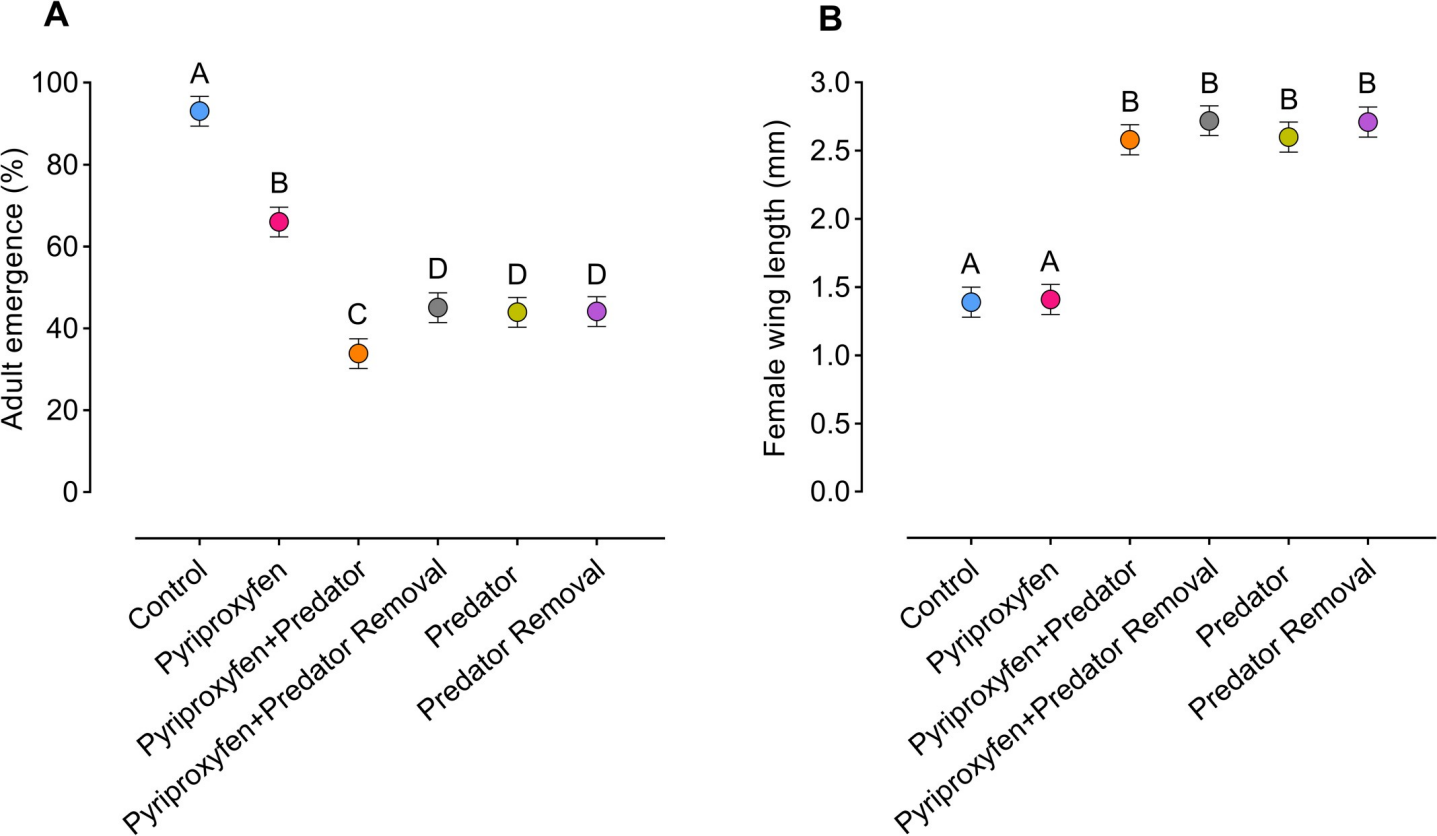
Effects of juvenile treatments on *Ae*. *aegypti* traits. (A) Adult emergence and (B) female wing length (an indicator of body size). Dots represent the means. Whiskers denote the standard error of the means. Statistical significance was determined by ANOVA. Different letters indicate significant differences (*p*<0.05) between juvenile treatment groups.

Analysis of variance showed significant juvenile treatment effects on adult emergence (F_5, 24_ = 557.4, *p* < .0001) (Fig 3A) and female wing length (an approximation of body size) (F_5, 24_ = 116.6, *p* < .0001) (Fig 3B). Control and pyriproxyfen treatments had significantly higher adult emergence (approximately 50% higher) compared to treatments involving reduced density or the presence of a predator (e.g., pyriproxyfen+predator, pyriproxyfen+predator removal, predator, predator removal treatments) (Fig 3A). Adult emergence was negatively related to wing length, indicating that treatments with high adult emergence (i.e., treatments contain high larval density) had shorter wings and treatments with low adult emergence (i.e., treatments contain low larval density) had longer wings, perhaps attributable to alterations in food and space. Wing lengths of adult females were significantly longer (approximately 45% longer) in treatments where larval density was reduced the most due to high mortality/removal (e.g., pyriproxyfen+predator, pyriproxyfen+predator removal, predator, predator removal treatments) and shorter in treatments with the highest adult emergence, including control and pyriproxyfen groups (Fig 3B).

### Zika virus infection, transmission, and viral titers in mosquitoes

**Fig 4:**
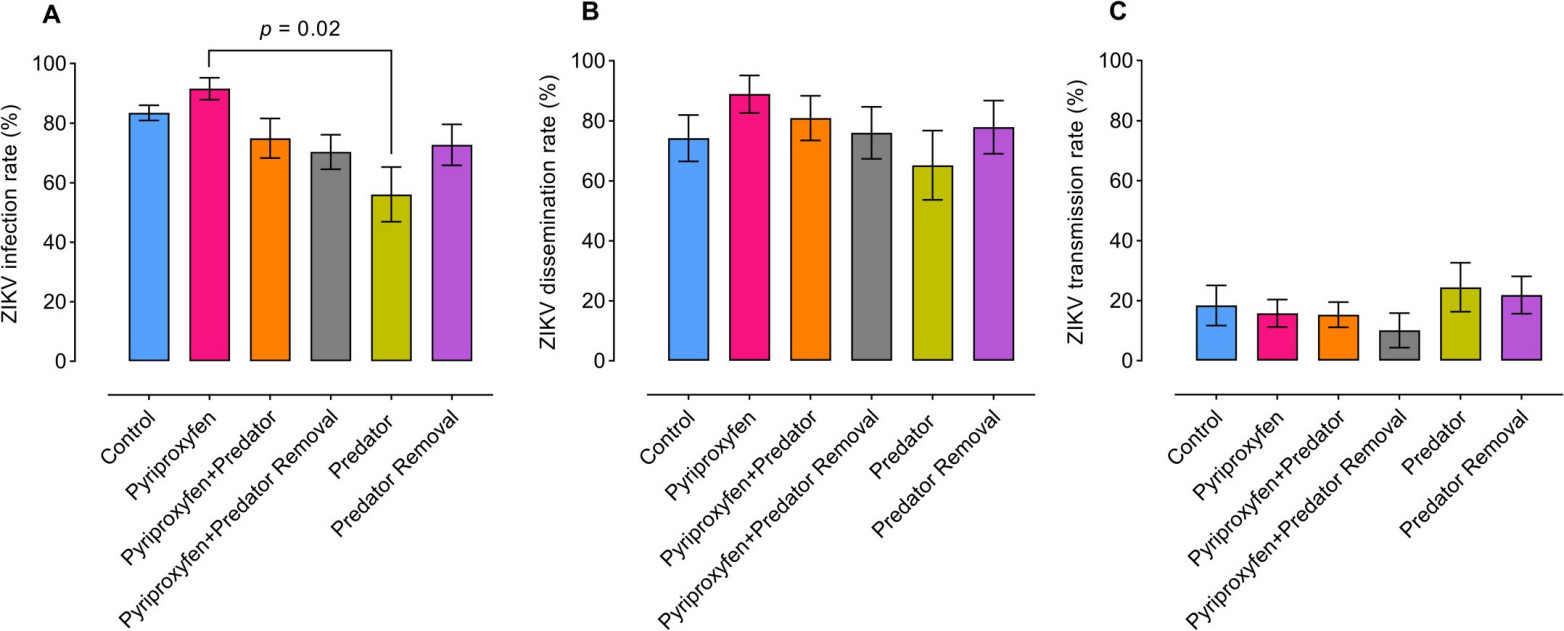
Effects of juvenile treatments on *Ae. aegypti*-ZIKV interactions. (A) Zika virus infection, (B) disseminated infection, and (C) saliva infection (transmission) were determined following orally exposure to ZIKV infectious blood meals. Bars represent the means. Whiskers denote the standard error of the means. For all treatments, viral infection, disseminated infection, and transmission were estimated at 15 days post-ZIKV infection. Control (*n* = 80), pyriproxyfen (*n* = 50), pyriproxyfen+predator (*n* = 47), pyriproxyfen+predator removal (*n* = 64), predator (*n* = 49), and predator removal (*n* = 63). Statistical significance was determined by ANOVA.

To evaluate the treatment-induced effects experienced by juvenile stages of *Ae*. *aegypti* on adult vector competence for ZIKV, a total of 353 adult females were tested for susceptibility to viral infection, disseminated infection, and saliva infection (transmission). Analysis of variance showed that treatment manipulations during juvenile stages have significant effects on susceptibility of adults to ZIKV infection, but only between pyriproxyfen and predator treatments (F_5, 24_ = 3.0, *p* = 0.02) (Fig 4A), whereas no significant effects observed between treatments in disseminated infection (F_5, 24_ = 0.6, *p* = 0.6) (Fig 4B), or transmission rates (F_5, 24_ = 0.7, *p* = 0.6) (Fig 4C). Adult mosquitoes exposed to *Tx*. *rutilus* predators during juvenile stages had lower susceptibility to ZIKV infection than other treatments. However, significant differences were observed only between predator and pyriproxyfen treatments (Fig 4A).

**Fig 5:**
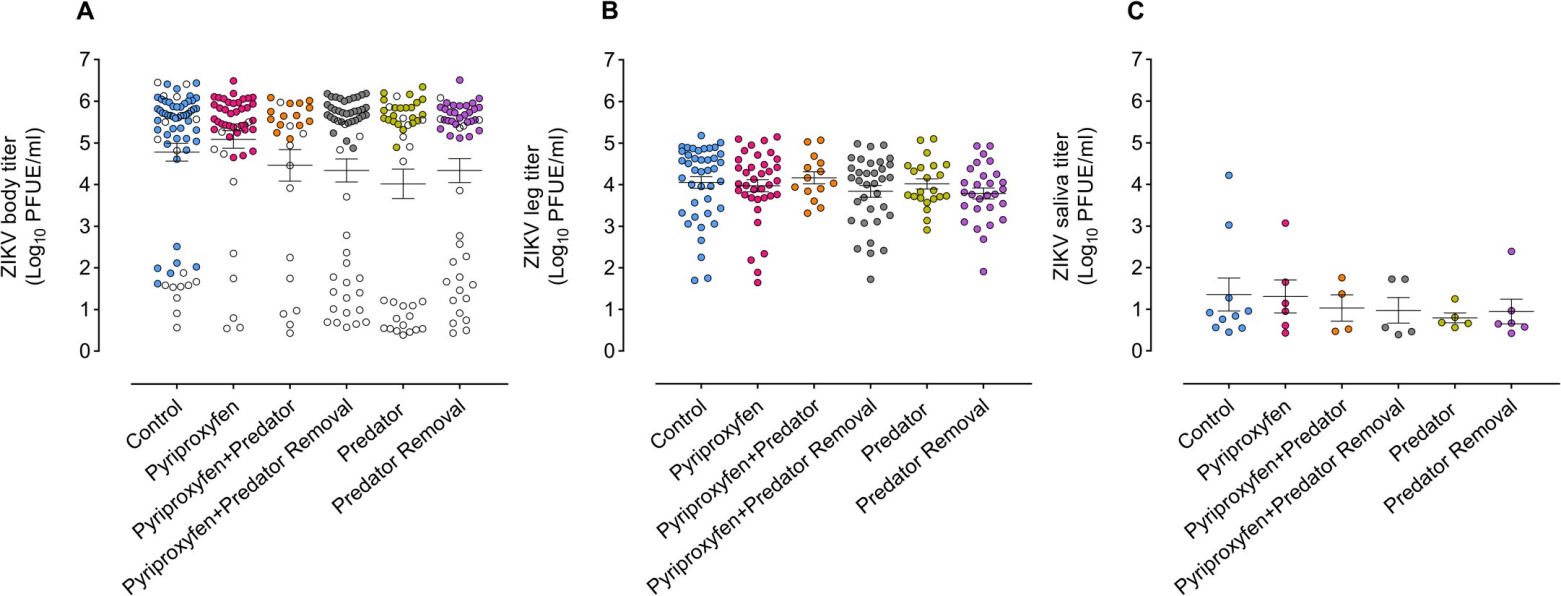
Effects of juvenile treatments on titrations of ZIKV in *Ae*. *aegypti* tissues. (A) Viral body, (B) leg, and (C) saliva titers (plaque forming unit equivalents/ml) of ZIKV-positive female *Ae*. *aegypti*. Horizontal lines indicate the mean of viral titers. Whiskers denote the standard error of the means. Each circle represents the titer for an individual female *Ae*. *aegypti*. Open circles in (A) represent the titers for females with non-disseminated infection of ZIKV (i.e., viral infection limited to mosquito midgut), whereas filled circles represent the titers for females with disseminated infection of ZIKV (i.e., viral dissemination from mosquito midgut epithelium). For all treatments, viral body, leg, and saliva titers were estimated at 15 days post-ZIKV infection.

Analysis of variance showed no significant differences between treatments in viral titers in mosquitoes’ tissues, including bodies (F_5, 24_ = 0.5, *p* = 0.7) (Fig 5A), legs (F_5, 24_ = 0.3, *p* = 0.8) (Fig 5B), and saliva (F_5, 24_ = 0.3, *p* = 0.8) (Fig 5C). Canonical-correlation analysis showed no significant canonical relationship between *Ae*. *aegypti* traits (adult emergence, size) and vector competence measurements (infection, disseminated infection, transmission) (Pillai's trace _6, 25_ = 0.3, *p* = 0.1).

## Discussion

Control measures that target juvenile mosquito stages can have both direct (mortality) and indirect effects that influence phenotypic traits of adults surviving exposure to control measures which may have crucial implications for mosquito-pathogen interactions. In this study, we investigated whether *Ae*. *aegypti* exposure to pyriproxyfen and predatory mosquito *Tx*. *rutilus* during the juvenile stage influence adult traits and susceptibility to infection and transmission of ZIKV. Our study demonstrated that the combination of pyriproxyfen and predators reduced adult emergence of *Ae*. *aegypti* more than observed in pyriproxyfen or predator treatment alone. To account for density reduction attributable to predation, we mimicked the daily mortality rate by predation in removal treatments to ensure that the indirect effects on mosquito traits reflected the absence of predator stress, but included density reduction (i.e., lower competition) since the latter has been found to alter traits, including susceptibility to arbovirus infection in mosquitoes [42,63]. Individuals that emerged to adulthood in treatments associated with lower density, including pyriproxyfen+predator, predator, and removals, were larger than mosquitoes from other treatments where density was higher and predator was absent (e.g., control and pyriproxyfen). Also, the combined effect of pyriproxyfen and predator exposure may be associated with changes in other traits among survivors that relate to body size. Female mosquito body size, a density dependent variable, is an important factor that may influence critical aspects of mosquito biology such as adult lifespan, blood feeding, fecundity, and mating success [59,64–66]. Large females of *Ae*. *aegypti* have the potential for lengthened adult lifespan, ingestion of larger blood meals and associated enhanced fecundity [64,59]. Our findings emphasize the importance of considering the interaction between environmental factors during juvenile stages due to control measures in arboviral disease epidemiology.

Control of juvenile stages of mosquitoes has primarily been achieved by targeting the aquatic habitats with traditional larvicides that induce mortality in the larval stages [67,68]. Unlike traditional larvicides (e.g., temephos and *Bacillus thuringiensis ssp*. *israelensis* (*Bti*)), pyriproxyfen has a unique mode of action that inhibits mosquito emergence to the adult stage, but does not kill the larvae. This mechanism introduces a delay in mosquito mortality which allows *Tx*. *rutilus* to further reduce the numbers of *Ae*. *aegypti* larvae before pyriproxyfen-induced mortality at the pupal stage. In our study, *Ae*. *aegypti* emergence to adulthood had a sharper reduction in pyriproxyfen+predator treatment compared to other treatments that contained either pyriproxyfen or predator alone. This result suggests that pyriproxyfen may be an attractive compound to use in vector control programs that integrates biological control agents such as *Tx*. *rutilus* against container inhabiting mosquitoes. Our observation supports a previous study reporting higher reduction in population of *Ae*. *aegypti* following the application of malathion in combination with naturally occurring or released *Tx*. *splendens* in Florida’s urban environments [69]. In our study, we did not observe inhibition of adult emergence in *Tx*. *rutilus* predators due to the application of pyriproxyfen, suggesting that the exposure to a low concentration of pyriproxyfen has no lethal effect on *Tx*. *rutilus*. Previous studies using *Toxorhynchites* spp. in combination with organophosphates [69,70] or *Bti* [71] found that susceptibility of *Toxorhynchites* spp. to these toxins is lower compared to *Ae*. *aegypti*, in part attributable to the larger size of *Toxorhynchites* spp. Additionally, the lethal dose of *Bti* in *Ae*. *aegypti* appeared to have negligible effects on later instar larvae of *Tx*. *rutilus* [71]. These findings suggest that the use of multiple juvenile sources of mortality (e.g., insecticide and biological control agents) can reduce pathogen transmission by inhibiting recruitment of adult vectors and associated population size.

The size of adult mosquitoes in our experiment varied between treatments. Treatments containing a predator or numerical reductions such as removal treatments produced larger individuals compared to other treatments where the predators were absent. It is likely that larval consumption attributable to predators and low density in prey removal treatments may accelerate development and enhance growth of surviving prey as they encountered less crowded conditions (i.e., release from competition), particularly in species that show no altered behavioral responses (i.e., reduce feeding activity) in the presence of predators [51,72,73]. Our results agree with other studies that observed larger sized individuals after emergence in the presence of dipteran predators or in prey removal treatments [54,55,74]. Although the exposure to chemical compounds such as malathion [75], spinosad [76], and *Bti* [77] during juvenile stages was associated with enhanced size of adults that survived exposure in *Ae*. *aegypti*, in our study, however, the sizes of adult survivors in pyriproxyfen treatment were relatively similar to those in control treatments, in which there was no pyriproxyfen or *Tx*. *rutilus*. One plausible explanation for the observed discrepancy in observations between studies is that insecticides (e.g., malathion, *Bti*, spinosad) with the modes of action that target the larval stage of mosquitoes and result in rapid death, which may allow larvae that survived exposure to experience competitive release from resources which can allow for enhanced growth and larger adults. In contrast, IGR like pyriproxyfen does not induce deaths in larval stages which may maintain larval competition and therefore diminish their growth as a density-dependent effect.

Several studies have detected both negative and positive relationships between mosquito body size and susceptibility to arboviral infection and transmission potential [78]. An infection study using La Crosse encephalitis virus (LACV) and *Ae*.* triseriatus* found that small females emerged from nutrient-deprived larvae had higher rates of infection, dissemination, and transmission of LACV following oral exposure to infectious blood meals compared to larger adults from well-nourished larvae [79]. In the same study, authors observed that midgut morphological variations (i.e., basement membrane thickness) between small and large females were associated with changes in vector competence for LACV. Similarly, small adults of *Ae*. *albopictus* emerged from crowded larval conditions had higher susceptibility to infection and disseminated infection of arboviruses such as DENV-2 and Sindbis virus (SINV) relative to large individuals from uncrowded larval conditions [63,80]. In contrast, larger *Ae*. *aegypti* mosquitoes were more likely to become infected with DENV-2 than medium or small mosquitoes derived from larval conditions that manipulated crowding and food availability [81]. Larger-sized field-captured *Ae*. *aegypti* adults were more likely to be infected with DENV than smaller conspecifics [82]. Along the same lines, *Ae*. *triseriatus* adults derived interspecific larval competition with superior competitor *Ae*. *albopictus* were larger and were associated with higher infection and dissemination rates of LACV compared to smaller individuals of *Ae*. *triseriatus* from intraspecific treatments [83]. Collectively, these results suggest that larval conditions may play an important role in mosquito-arbovirus interactions and that size alone may not be causally connected to alterations in vector competence. In our study, we did not find an overall relationship between mosquito body size and ZIKV vector competence measurements, suggesting that mosquito size *per se* is not necessarily a factor influencing *Ae*. *aegypti* vector competence for ZIKV. Our findings are consistent with previous studies that found no association between mosquito size and infection and disseminated infection of SINV [75] and DENV-1 [84].

Juvenile environmental factors such as nutritional deprivation and intra- or interspecific competition are stressors that can induce changes in subsequent adult life- history traits [78], immune function [85,86], and associated susceptibility to infection with pathogens [63,78,80,87,88]. In the present study, exposure to a predator alone reduced susceptibility to ZIKV infection in subsequent adults relative to pyriproxyfen treatment, suggesting that predator stress may influence adult *Ae*. *aegypti* interactions with ZIKV, primarily acting at the initial site of infection. Although the presence of predator had no direct effect on ZIKV disseminated infection and transmission in *Ae*. *aegypti*, sublethal effects of predation stress may reduce fecundity and lifespan of adults, traits which influence pathogen transmission [53,54].

In our study, we hypothesized that larvae exposed to predation stress would show reduction in body size in comparison to their conspecific from removal treatments where predators were absent. We found that adults from larval treatments containing a predator (e.g., pyriproxyfen+predator and predator) had similar body size compared to conspecifics in removal treatments, suggesting that *Ae*. *aegypti* did not exhibit anti-predator behavior (i.e., reduction in feeding activity and longer development time which can produce smaller adults) [54,72,89]. In contrast, adults of malaria mosquito *An*. *coluzzii* showed smaller body sizes (shorter wings) at metamorphosis in the presence of a predatory backswimmer [53]. Similarly, a field study showed smaller sized *Ae*. *triseriatus* mosquitoes from tires with *Tx*. *rutilus* compared to containers without *Tx*. *rutilus*, perhaps attributable to reduced movement, food intake and size at metamorphosis [90]. Collectively, these results suggest that species-specific anti-predator behavior among mosquitoes likely influences net growth.

Different responses to viral pathogen infection following exposure to insecticides have been observed in mosquito vectors [78,91]. Interestingly, mosquitoes exposed to pyriproxyfen during juvenile stages, in our study, had enhanced susceptibility to ZIKV infection, but not disseminated infection or transmission, only when compared to the predator treatment. The reason for the lack of significant differences among other treatments is unclear, but it may possibly reflect differences in immune responses early during infection attributable to the juvenile environment. Previous studies showed that juvenile mosquito exposure to *Bti* did not significantly affect the adult vector competence for arboviruses, including DENV-1 and CHIKV [79,91]. Additionally, adult mosquito exposure to pyrethroid bifenthrin incorporated with sugar solution showed no alterations in ZIKV infection, disseminated infection, or viral titers after 14 days post-infection [92]. These outcomes suggest that insecticide-induced stress experienced during either juvenile or adult stages may have minimal influence in altering mosquito vector competence for arboviruses. On the contrary, enhancement in susceptibility of mosquitoes to infection with arboviruses, including DENV and SINV was previously observed following insecticide exposure [41,75,93]. Taken together these observations suggest that exposure to insecticides may cause enhancement or cause no effect on mosquito susceptibility to infection with pathogens. These contrasting consequences underline the need to continue assessing the sublethal effects of insecticides exposure to understand their subtle effects on mosquito-pathogen interactions rather than just focus on their lethal mortality. Future studies should consider the influence of sublethal exposure of insecticides and predators on mosquito immune responses to pathogens and additional phenotypic traits of adults that are likely to contribute to vectorial capacity, including host seeking and blood feeding behaviors, reproduction, and adult lifespan.

Our data demonstrate that combined effects of pyriproxyfen and predator *Tx*. *rutilus* have potential to reduce ZIKV transmission through high level of adult *Ae*. *aegypti* emergence inhibition which can lead to greater reduction in mosquito population size. However, surviving individuals may have altered traits such as greater net growth and size at emergence, potentially enhancing fitness. Our study underscores the importance of measuring the consequences of abiotic and biotic interactions occurs during juvenile stages due to mosquito control practices on mosquito-pathogen interactions as related to public health protection.
